# False hyperthyroidism caused by interference in immunoassays

**DOI:** 10.1515/almed-2020-0097

**Published:** 2020-11-13

**Authors:** Clara Jiménez García, Piedad Ortega Fernández, María Eugenia Torregrosa Quesada, Victoria González Bueno, María Teresa Botella Belda, Rocío Alfayate Guerra

**Affiliations:** Laboratory of Hormones, Hospital General Universitario de Alicante, Pintor Baeza 11, Alicante, Spain; Laboratory of Clinical Chemistry, Hospital General Universitario de Elche, Almazara, Elche, Spain

**Keywords:** heterophilic antibodies, hyperthyroidism, interference of immunoassay

## Abstract

**Objectives:**

Immunoassays used to assess thyroid function are vulnerable to different types of interference that may affect clinical decision-making.

**Case presentation:**

We report the case of a 37-year-old woman who developed iatrogenic hypothyroidism after having received radioiode therapy who visited our hospital for her annual checkup. The patient was asymptomatic, without signs suggestive of thyroid disease. However, laboratory analysis proved otherwise: thyrotropin (TSH) 7.75 mU/L, thyroxine (FT4) >7.7 ng/dL.

**Conclusions:**

The inconsistency between her clinical symptoms and the biochemistry data raised the possibility of a methodological interference. A thorough evaluation of the main causes of interference was conducted in the laboratory to exclude the presence of interference in TSH and FT4. Finally, different interfering agents were identified, which affected free thyroid hormone and TSH determination.

## Introduction

The parameters used to assess thyroid function include thyrotropin (TSH), free thyroxine (FT4) and free triiodothyronine (FT3). In some settings, the relationship between TSH and thyroid hormones does not fit with the expected feedback mechanisms. This may be due both to a pathophysiological process and to methodological interference. The immunoassays used to assess thyroid function are vulnerable to different types of interferences that may affect clinical decision-making [1].

## Case presentation

A 37-year-old woman treated with levothyroxine who developed iatrogenic hypothyroidism after having received radioiodine therapy was seen in our hospital (laboratory 1) for her annual checkup.

The patient was asymptomatic, without signs suggestive of thyroid disease. Her biochemistry demonstrated: TSH 7.75 mU/L (reference value [RV]: 0.38–4.84), FT4>7.7 ng/dL (RV: 0.8–1.8) and FT3 of 7.6 pg/mL (RV: 1.8–4.6 pg/mL). The three parameters were measured using an automated electrochemiluminescent immunoassay analyzer (Cobas 8000® [Roche Diagnostics^®^]). The euthyroid state of the patient was confirmed by determination of sex hormone-binding globulin, which was 38.7 nmol/L (RV: 11–124).

The presence of elevated concentrations of thyroid hormones, normal or elevated TSH levels, the absence of clinical symptoms of hyperthyroidism and in the absence of pharmacological interferences raised the possibility of a methodological interference. The main causes of interference in TSH and FT4 determination were evaluated including macro-TSH, heterophile antibodies, thyroid autoantibodies, biotin, antistreptavidin or antiruthenium antibodies [2], [3], [4], [5], [6], [7]. The presence of interferences was assessed using the following protocol [8]:

1) Results were confirmed by repeating the test in the same sample using the same method to discard a problem with pipetting, inadequate washing or the presence of aggregates or bubbles.
2) Measurements were repeated in a new sample from the same patient, which yielded the same results.
3) Measurements were repeated using a different method. For this purpose, (in this regard) the sample was sent to other laboratories where a different analyzer was used. The methods used and results obtained are shown in Table 1.
4) Serial dilutions were performed to measure TSH. Dilutions of the sample were performed using the diluent recommended by the manufacturer (1:2, 1:4, 1:8). No loss of linearity was observed in serial dilutions.
5) The presence of macro-TSH was excluded by precipitation with polyethylene glycol (PEG 6000) at 20%, with a recovery percentage of 114% (acceptable range: 80–120%).
6) Rheumatoid factor (RF) was measured, with a result of <20IU/mL (RV: <20IU/mL).
7) The presence of heterophile antibodies was assessed by incubation of the sample in heterophilic blocking tubes (HBT tubes, Scantibodies^®^) (Table 2).
8) Tests for antimicrosomal antibodies and anti-TSH receptor antibodies were negative.
9) The sample was finally sent to the Department of Research and Development of Roche Diagnostics.

**Table 1:** Methods and results obtained in the different laboratories.

Laboratory	System (manufacturer)	TSH, mU/L	FT4, ng/dL
1	Cobas (Roche)	7.7	>7.7
2	Vitros (Ortho)	23.7	0.8
3	Arquitect (Abbott)	49.1	0.6

TSH, thyrotropin; FT4, free thyroxine.

**Table 2:** Results obtained before and after the samples were processed in HBT tubes.

Tests	Laboratory 1 (Roche-Cobas)	Laboratory 2 (Ortho-Vitros)	Laboratory 3 (Abbott-Arquitect)
TSH, mU/L	7.7	23.7	49.1
FT4, ng/dL	>7.7	0.8	0.6
**Scantibodies**			
TSH, mU/L	7.5	20.7	55.4
FT4, ng/dL	1.0	0.7	0.7

HBT, heterophilic blocking tubes; TSH, thyrotropin; FT4, free thyroxine.

## Discussion

Immunoassays are the most widely used methods to assess thyroid function. However, interference may occur, and false results in the form of overestimation or underestimation may be obtained. When undetected, interference may lead to misdiagnosis, ineffective treatment and poor clinical outcomes [2], [ 3].

In our case, once the potential preanalytical and physiopathological causes were excluded, we posited that inconsistency between TSH and FT4 levels could be due to a methodological interference.

The repetition of determinations in analyzers that use a chemiluminescent immunoassay (Ortho-Vitros and Abbott-Arquitect) with different antibodies confirmed our suspicion of a methodological interference, as different results were obtained according to the method employed. In our laboratory, TSH was measured by a method based on chimeric antibodies, with lower occurrence of interferences. All methods yielded different results, which suggest a potential interference that may affect all or two of the methods.

FT4 determination was performed by a one-step competitive method in laboratories 1 and 2 and by a two-step method in laboratory 3. Two-step methods are less subject to interference. The repetition of tests in an analyzer based on a different technique is useful in the determination of conflicting thyroid hormone levels. The FT4 result in laboratories 2 and 3 confirmed that the FT4 result obtained with our method was subject to interference.

The presence of an interfering agent may cause lack of linearity in serial dilutions. Its utility is limited, as only 40% of samples with known endogenous antibodies show lack of linearity [9]. Moreover, it is not applicable to all analytes. Therefore, this test should not be employed alone to assess the result of an immunoassay or exclude the presence of interfering endogenous antibodies. In our case, a loss of linearity was not observed in serial TSH dilutions. This test cannot be used for FT4 since a free hormone test does not allow dilution because it alters the balance between hormones and binding proteins.

Macro-TSH derives from the binding of TSH to an immunoglobulin, which produces an immunocomplex that may interfere with TSH determination.

The presence of macro-TSH leads to falsely elevated results, depending on the antibodies used in immunoassays. With PEG, a precipitate of high-molecular-weight complexes is obtained. A precipitate of the sample was obtained using PEG in all laboratories to confirm the absence of macro-TSH, as a similar recovery rate was observed in all samples.

RF includes a heterogeneous group of antibodies targeted against antigenic determinants of the Fc region of IgG molecules. Patients with rheumatoid arthritis or other autoimmune diseases may exhibit elevated RF levels. RF can bind to the antibodies of the reagent and yield falsely elevated levels [10]. In our case, RF interference was excluded, as it was undetectable.

Heterophile antibodies are human antibodies targeted against immunoglobulins of other animal species that may cause method-specific interference. The prevalence of these antibodies has increased as a result of the therapeutic and diagnostic use of monoclonal antibody-based biological agents [11]. The estimated incidence of heterophile antibody interference ranges from 0.04 to 4% [12], [ 13], although manufacturers add antibody-based blockers to immunoassay reagents to reduce interference. Since the complete removal is not always possible, heterophile antibody-based blockers can be useful as they contain specific binding agents that inactivate heterophile antibodies. Nevertheless, these blockers are dependent upon the method and the technique employed. In our case, the presence of heterophile antibodies was assessed by the scantibodies HBT system, which is suitable for our platform. After the sample was processed with the blocking agent, heterophile antibody interference was confirmed in the FT4 assay in laboratory 1, as once they were blocked, results were normalized and became similar to those obtained in the other two analytical platforms.

As biotin at high doses (>5 mg/day) may interfere in immunoassays that use streptavidin-biotin binding to amplify the detection signal, both in competitive (FT4) and noncompetitive methods (TSH), this may cause a positive or negative interference, respectively [14]. In Roche, interference may occur in TSH, FT4 and FT3 determinates due to excess biotin. In Ortho, only TSH is affected, as the techniques used to measure FT4 and FT3 are biotin free. The Abbott immunoassay is not based on the streptavidin-biotin binding system; therefore, it proves to be the method of choice to indirectly identify biotin interference [2]. Our patient reported not taking any biotin supplementation.

Antiruthenium antibodies are released in the presence of high levels of environmental ruthenium [15]. In 2006, Roche improved its reagents, although interference was not eliminated completely. However, in 2020, TSH, FT4 and FT3 reagents were reformulated and interference has been eliminated.

To complete the study and perform a more thorough evaluation, the sample was sent to the Department of Research and Development of Roche Diagnostics. The results obtained did not demonstrate biotin or antiruthenium antibody interference, although the presence of an unspecific interference was confirmed.

A limitation of this study is that inconsistency in TSH levels obtained in each laboratory could not be explained, although the interference that causes conflicting results cannot always be clarified. This is a complex setting that includes different interfering factors that affect free thyroid hormones and TSH determination.

## Conclusions

When results are inconsistent, consider the presence of chemical interfering factors associated with automated immunoassays. It is important to be familiar with the platform to identify the etiology of interference. However, the interfering agent cannot always be identified.

It is the laboratories’ responsibility to identify and report the presence of interference to minimize its impact on clinical decisions. Continuous feedback between laboratorians and clinicians and manufacturers is essential to identify and prevent interference.
